# Differences in prescribing and dispensing at end-of-life between people who died at home before and during the Covid-19 pandemic in Scotland.

**DOI:** 10.23889/ijpds.v9i5.2787

**Published:** 2024-09-18

**Authors:** Jan Savinc, Iain Atherton

**Affiliations:** 1 Edinburgh Napier University; 2 Scottish Centre for Administrative Data Research

## Objectives

To compare levels of palliative care need that were met through prescriptions and dispensing of medicines at end-of-life for people who died at home in Scotland during and prior to the Covid-19 pandemic.

## Approach

Retrospective observational study of linked routinely collected data: death registrations in Scotland during 12-month pandemic period (starting 23rd March 2020) and pre-pandemic period (5 years prior), linked to inpatient hospital admissions and community prescribing & dispensing data. Prescribing/dispensing will be summarised for pandemic & pre-pandemic cohorts and palliative care needs estimated using death registrations and inpatient admissions.

## Results

An estimated N=59,152 individuals died with palliative care needs during the first year of the pandemic, or approximately 11% more than in 2019/20. Community prescribing & dispensing will be compared between the pandemic and pre-pandemic periods as a proxy measure of primary care use and to investigate shortages. Anticipatory medication use will be compared to estimate quality of death at home for decedents.

## Conclusions

Deaths at home increased by about a third during the first year of the pandemic in Scotland and continue at similar levels currently. Understanding the effect of the pandemic on prescriptions and dispensing at end-of-life will help explain the shift of deaths to home settings in addition to demographic and service contact changes early in the pandemic.

## Implications

Studying the changes in end-of-life medication use will inform policy to better continue supporting people dying at home, especially where there are unmet needs or gaps between prescribed and dispensed medications.

